# Diagnostic challenge of paroxysmal sympathetic hyperactivity (PSH) associated with diffuse axonal injury (DAI) in head trauma

**DOI:** 10.1186/2193-1801-3-752

**Published:** 2014-12-18

**Authors:** AM Bueno González, MC Corcobado Marquez, M Portilla Botelho, A Ambrós Checa

**Affiliations:** Critical Care Unit, Hospital General Universitario Ciudad Real, C/Obispo Rafael Torija s/n, 13005 Ciudad Real, Spain

Dear Editor it is well known that many patients show a significant altered level of consciousness after head trauma, and they subsequently present neurological sequelae, without any relevant findings in the CT scan. This is often due to the presence of diffuse axonal injury (DAI). The purpose of this study was to investigate the factors associated with DAI, especially the occurrence of paroxysmal sympathetic hyperactivity (PSH). We performed a retrospective analysis of 189 patients admitted in Intensive Care Unit (ICU) with severe traumatic brain injury, from 2008 to 2012. DAI was defined as a GCS score of 8 or less, lasting for more than 6 hours, with a normal CT or with small hemorrhages (<10 mm) in the CT and/or MRI (Chelly et al. 2011).

The incidence of DAI was 28%. Factors associated with a poor prognosis were, ≥ 5 hemorrhagic millimeter lesions, lesions in *corpus callosum* and/or in brain stem (*p* = 0.004. RR: 2.40), a motor GCS ≤3 at admission (*p* = 0.002. RR: 1.97) and the development of paroxysmal sympathetic hyperactivity (PSH) (*p* = 0.007. RR: 2).These patients did not recover the level of consciousness at ICU discharge, although there was no relationship with mortality. The results of the multivariate logistic regression between risk factors and DAI, are summarized in Table 1. In this model, higher energy trauma, especially car traffic accidents and intraventricular hemorrhage were independent predictors of DAI. Epidural hematomas and subdural hematomas were “protective” factors as their presences meant a lower risk of DAI. A plausible explanation is the different mechanism of injury: extraaxial hematomas are related to trauma caused by a direct impact and axonal injury is related to acceleration and deceleration forces (Calvi et al. 2011).

**Table 1:** **Multivariate logistic regression analysis of the association between risk factors and diffuse axonal injury** (**DAI)**

Factors	OR	95% CI	***p***
Higher energy traffic accidents	3.690	1.556-8.737	0.003
Hemorrhagic Contusion	0.549	0.223-1.348	0.549
Subdural Hematoma	0.378	0.162-0.885	0.025
Epidural Hematoma	0.083	0.016-0.417	0.003
Subarachnoid Hemorrhage	1.182	0.506-2.762	0.700
Intraventricular Hemorrhage	9.133	3.477-23.995	0.000

CI, confidence interval.

One of the worst outcome factors of DAI is PSH but it often remains unidentified due to the complexity of the diagnosis. Patients with DAI frequently develop this kind of crises and they are often misinterpreted. In our study the incidence of PSH was 19%, whereas the reported incidence varies from 8% and 33% (Lv et al. 2011). This broad variation is mainly originated from different inclusion criteria and definitions of PSH. Most studies are based in Baguley’s *et al.* definition (Baguley et al. 1999). A diagnosis of PSH required at least one daily paroxysm occurring for at least 3 consecutive days, and the exclusion of other causes such as withdrawal syndrome, epileptic seizures, and sepsis (Hörtnagl et al. 1980).

The diagnostic process has often been a significant challenge. Recently, an international and multidisciplinary consensus document was published (Baguley et al. 2014), aiming to i) to develop a conceptual definition; ii) to resolve confusion regarding the nomenclature, iii) to produce a consensus set of diagnostic criteria and iv) to develop a diagnostic tool for evaluating the presence and severity of the crisis. In this document the name of paroxysmal sympathetic hyperactivity (PSH) was proposed and was defined as “A recognized syndrome, in a subgroup of survivors of severe acquired brain injury, which involves simultaneous, paroxysmal transient increases in sympathetic (elevated heart rate, blood pressure, respiratory rate, temperature, sweating) and motor (posturing) activity’. This process also developed a diagnostic tool consisting of two components*:* the probability of diagnosis (the Diagnosis Likelihood Tool [DLT]) and the severity of the clinical features (the Clinical Feature Scale [CFS]). The numerical output of these two components is added together to estimate the diagnostic likelihood of PSH. These components form the PSH Assessment Measure (PSH-AM) (Baguley et al. 2014). To date, this is the first scale that standardizes published diagnostic criteria and assesses the severity of the crisis. It is easy to apply in clinical practice and very useful to improve the efficiency of diagnosis. While we wait for preliminary results and validation studies, this consensus document solves what until now was a real diagnostic challenge.
